# Prospective Observational Study of Polynucleotide Injections for Periorbital Rhytides

**DOI:** 10.1111/jocd.70736

**Published:** 2026-02-13

**Authors:** Georges Ziade, Elias Keyrouz, Joy El Maalouf, Dana Swaidan, Desiree Karam, Dayane Daou, Georges Fidawi

**Affiliations:** ^1^ Department of Otolaryngology‐Head and Neck Surgery Lebanese American University School of Medicine Beirut Lebanon; ^2^ Faculty of Medicine Holy Spirit University of Kaslik Kaslik Lebanon; ^3^ Department of Anesthesia American University of Beirut Medical Center Beirut Lebanon; ^4^ Department of Dermatology American University of Beirut Medical Center Beirut Lebanon

## Abstract

**Introduction:**

Polynucleotides (PN), derived from salmon sperm DNA, are highly purified DNA fragments with reported tissue‐repair and anti‐inflammatory properties mediated through adenosine A2A receptor activation. These agents have been increasingly used in aesthetic dermatology for skin rejuvenation and scar treatment. This study aimed to evaluate longitudinal changes in periorbital appearance and patient‐reported outcomes following intradermal polynucleotide injections.

**Methods:**

This prospective observational case series included patients presenting for undereye rejuvenation who received intradermal injections of absorbable polynucleotides (7.5 mg/mL; Pluryal Silk, MD Skin Solutions, Luxembourg), with a total volume of 2 mL per session. Outcomes were assessed using validated FACE‐Q patient‐reported questionnaires at baseline and at 1, 3, and 6 months following treatment. Adverse events were assessed 2 days after each treatment session. Statistical analysis was performed using paired *t*‐tests.

**Results:**

Forty‐two patients were included. Significant improvements in lower eyelid and crow's feet appraisal scores were observed at all follow‐up time points compared with baseline (*p* < 0.001). The greatest improvements and highest patient satisfaction were observed at 3 months following two treatment sessions. Reported adverse events were minimal.

**Conclusion:**

In this prospective observational cohort, intradermal polynucleotide injections were associated with improvements in periorbital appearance and patient‐reported satisfaction over baseline, with minimal adverse events. These findings support further controlled studies to confirm efficacy and refine treatment protocols.

## 1 Introduction

Polydeoxyribonucleotides (PDRN), derived from salmon sperm DNA, are low‐molecular‐weight DNA fragments that exert anti‐inflammatory and tissue‐repair effects through activation of adenosine A2A receptors. Polynucleotides (PN), which are high‐molecular‐weight derivatives from the same source, exhibit longer‐lasting effects due to their increased viscosity. These biopolymers are thought to stimulate fibroblast proliferation, collagen synthesis, and angiogenesis, thereby enhancing skin texture and elasticity. Their use in aesthetic medicine has surged due to their ability to stimulate natural skin‐regeneration processes without relying on synthetic or cross‐linked fillers [1].

Recent advances have highlighted the synergistic potential of PN when combined with non–cross‐linked hyaluronic acid (HA), particularly in sensitive areas such as the periorbital region. Abuyousif et al. [2] demonstrated that PN‐HA complexes not only improve periorbital skin quality and hydration but also exhibit regenerative effects by enhancing extracellular matrix (ECM) remodeling and fibroblast viability in vitro. Additionally, a prospective randomized study by Araco et al. [3] showed that PN‐HPT combined with cross‐linked HA provided superior wrinkle reduction and dermal restructuring compared with HA alone in the treatment of moderate to severe nasolabial folds, further supporting PN's biorevitalizing potential. These findings underscore the biostimulatory value of PN for treating delicate regions such as the lower eyelid, where conventional fillers may pose higher risks of edema, irregularities, or Tyndall effect.

Several clinical studies have further supported the benefits of PN in various dermatologic and cosmetic indications. In the treatment of atrophic acne scars and facial photoaging, PN has demonstrated efficacy in improving skin tone, firmness, and hydration while reducing fine lines and wrinkles [4]. Furthermore, PN's high biocompatibility and favorable safety profile make it suitable for sensitive areas such as the periorbital region, where the skin is thinner and more prone to adverse effects from conventional treatments. Highly purified polynucleotides (PN‐HPT) have also emerged as a valuable biostimulatory option, offering a non‐cross‐linked alternative for facial rejuvenation and skin priming [5].

Despite their increasing use, real‐world evidence on PN application specifically for undereye wrinkles remains limited [6]. This study aimed to evaluate the clinical efficacy, patient satisfaction, and tolerability of intradermal polynucleotide injections in treating undereye wrinkles and crow's feet over a 6‐month period.

## 2 Materials and Methods

This prospective case series evaluated adults seeking undereye rejuvenation at the senior author's aesthetic dermatology clinic. Participants were consecutively recruited from patients presenting for elective undereye rejuvenation between January and June 2024. All individuals who met inclusion criteria and provided written informed consent were enrolled.

Inclusion criteria were adults aged 25–65 years with visible periorbital rhytides (Glogau II–III) and Fitzpatrick skin types I–IV. Exclusion criteria included prior periorbital treatments (e.g., botulinum toxin, fillers, lasers, mesotherapy, biostimulators) within the past 12 months, active infections, coagulopathies, pregnancy, lactation, a history of hypertrophic scarring, residual superficial hyaluronic acid fillers, and allergy to fish.

A total of 42 patients underwent intradermal injections of highly purified polynucleotides (Pluryal Silk, 7.5 mg/mL; 2 mL total per session) using a 30G needle under sterile conditions. The injected volume was distributed evenly as 1 mL per side in the periorbital region. The product was injected intradermally along wrinkle lines to achieve a tenting effect. Topical anesthetic cream (lidocaine‐based) was applied prior to injection at the discretion of the treating physician and removed before treatment. Each patient received two treatment sessions spaced 3 weeks apart, with a third session performed for those with persistent moderate‐to‐severe wrinkles following the second session.

Patients completed FACE‐Q Satisfaction modules, which are validated instruments designed for cosmetic outcome assessment and known for high reliability and sensitivity to aesthetic changes. FACE‐Q appraisal and satisfaction scales are transformed to scores ranging from 0 to 100. For appraisal scales, higher scores indicate greater perceived severity of wrinkles, whereas for satisfaction scales, higher scores reflect greater satisfaction with outcomes and decision‐making. The Recovery Early Symptoms module was administered by phone 2 days after each treatment session. The Appraisal of Lower Eyelids and Crow's Feet Lines questionnaires were collected at baseline and at 1, 3, and 6 months. Satisfaction with Outcome and Satisfaction with Decision modules were administered at 1, 3, and 6 months. Baseline assessments were considered pre‐treatment; the 1‐month assessments captured outcomes following the first session; the 3‐month assessments reflected early outcomes after two sessions; and the 6‐month assessments were collected prior to the follow‐up or maintenance session to represent longer‐term results (see Appendix A).

The primary outcome was the change in FACE‐Q lower eyelid and crow's feet appraisal scores from baseline to follow‐up assessments at 1, 3, and 6 months. Secondary outcomes included FACE‐Q Satisfaction with Outcome and Satisfaction with Decision scores, as well as treatment‐related adverse events.

Standardized two‐dimensional photographs were obtained using a Canon EOS 80D camera mounted on a fixed‐height Manfrotto tripod. Camera settings (ISO, aperture, focal length, and white balance) were kept constant across all sessions. Lighting was standardized using two continuous LED softboxes positioned at 45° angles. Patients were positioned at a fixed distance (1.2 m) and instructed to maintain a neutral facial expression. Images were reviewed by two independent physicians not involved in treatment administration. Due to the recognizability of the periorbital region, assessor blinding was not feasible; however, evaluators were not informed of the assessment time points. All clinical photographs were obtained without the use of digital filters or post‐processing beyond standard cropping. Written informed consent for the use and publication of identifiable clinical photographs was obtained from all participants.

Descriptive statistics (mean ± SD) and paired *t*‐tests were used to assess changes over time, with significance set at *p* < 0.05. Statistical analyses were performed using SPSS Version 27 (IBM Corp., Armonk, NY). Prior to analysis, FACE‐Q score distributions were visually inspected and assessed for normality and were found to be approximately normally distributed, supporting the use of parametric paired *t*‐tests. Given the exploratory, hypothesis‐generating nature of this prospective case series, no formal correction for multiple comparisons was applied, as such adjustments may increase the risk of type II error and obscure potentially meaningful clinical trends. Effect sizes (Cohen's *d*) were calculated for key within‐subject comparisons using pooled standard deviations to estimate the magnitude of observed changes. All 42 participants completed baseline and 1‐month assessments; 40 (95.2%) completed the 3‐month assessment, and 38 (90.5%) completed the 6‐month assessment. Missing data were handled using pairwise deletion for each comparison. As this was an exploratory prospective case series designed to generate preliminary clinical data, no a priori power calculation was performed.

All participants provided written informed consent, and all data were anonymized. Ethical approval for this study was obtained from the Institutional Review Board of the Lebanese University. Pluryal Silk was used exclusively to maintain protocol consistency, and the authors declare no financial, advisory, or commercial relationship with the manufacturer.

## 3 Results

The study included 42 patients, with a mean age of 43.55 years (SD ± 6.34); 73.8% were female, and 38.1% were current or occasional smokers (Table 1). Two days after the first injection, adverse events were assessed using the FACE‐Q Recovery Early Symptoms module. The most frequently reported symptoms were mild swelling and discomfort, with a few instances of minor bruising, indicating excellent short‐term tolerability. During subsequent follow‐up visits at 1, 3, and 6 months, no delayed or persistent adverse events were observed, including nodules, prolonged edema, Tyndall‐like discoloration, infection, or other treatment‐related complications. Follow‐up completion rates were high, with 40 participants completing the 3‐month assessment and 38 completing the 6‐month assessment.

**TABLE 1:** Baseline demographics and descriptive variables (Total *N* = 42).

Variable	*N*	%
Gender (Male)	11	26.2
Smoking status (yes)	16	38.1

B – continuous variables			
Variable	Mean	SD	95% Confidence interval
Age (years)	43.55	6.34	3.51–4.73
AEs at 2 days score	20.67	2.01	3.07–4.60
Baseline Lower Eyelids	20.50	1.61	
Baseline crow's Feet	21.88	3.14	
1 month lower eyelids	13.95	2.46	
1 month crow's feet	15.12	3.39	
1 month satisfaction outcome	17.48	2.71	
1 month satisfaction decision	18.07	4.22	
3 months lower eyelids	10.98	1.39	
3 months crow's feet	11.31	2.88	
3 months satisfaction outcome	21.60	2.66	
3 months satisfaction decision	21.90	3.40	
6 months lower eyelids	12.95	1.64	
6 months crow's feet	14.45	2.06	
6 months satisfaction outcome	18.45	0.77	
6 months satisfaction decision	19.69	2.93	

Significant improvements were observed in the FACE‐Q Satisfaction with Outcome and Satisfaction with Decision scores at 1, 3, and 6 months compared with baseline (Table 2). Mean Satisfaction with Outcome scores increased from 17.48 ± 2.71 at 1 month to 21.60 ± 2.66 at 3 months and remained higher at 18.45 ± 0.77 at 6 months. Mean Satisfaction with Decision scores increased from 18.07 ± 4.22 at 1 month to 21.90 ± 3.40 at 3 months and remained elevated at 19.69 ± 2.93 at 6 months (see Table 3).

**TABLE 2:** Mean differences in Face‐Q appraisal lower eyelids and crow's feet lines (Baseline, 1, 3, and 6 months).

Variable	Mean difference	*p*	95% Confidence interval
Baseline vs. 1 month Lower eyelids	6.548	< 0.001	8.78–10.27
Baseline vs. 1 month crow's feet	6.762	< 0.001	9.24–11.90
Baseline vs. 3 months Lower eyelids	9.524	< 0.001	7.11–7.99
Baseline vs. 3 months crow's feet	10.571	< 0.001	6.17–8.69
Baseline vs. 6 months Lower eyelids	7.548	< 0.001	
Baseline vs. 6 months crow's feet	7.429	< 0.001	
1 month vs. 6 months Lower eyelids	1.00	0.057	
1 month vs. 6 months crow's feet	0.667	0.173	
3 months vs. 6 months Lower eyelids	−1.976	< 0.001	
3 months vs. 6 months crow's feet	−3.143	< 0.001	

**TABLE 3:** Comparison between 1 month, 3, and 6 months regarding Face‐Q Satisfaction with Outcome and satisfaction with decision.

Variable	Mean difference	*p*
1 month vs. 3 months Satisfaction Outcome	−4.119	< 0.001
1 month vs. 3 months Satisfaction Decision	−3.833	< 0.001
1 month vs. 6 months Satisfaction Outcome	−0.976	0.014
1 month vs. 6 months Satisfaction Decision	−1.619	< 0.001
3 months vs. 6 months Satisfaction Outcome	3.143	< 0.001
3 months vs. 6 months Satisfaction Decision	2.214	< 0.001

**FIGURE 1:**
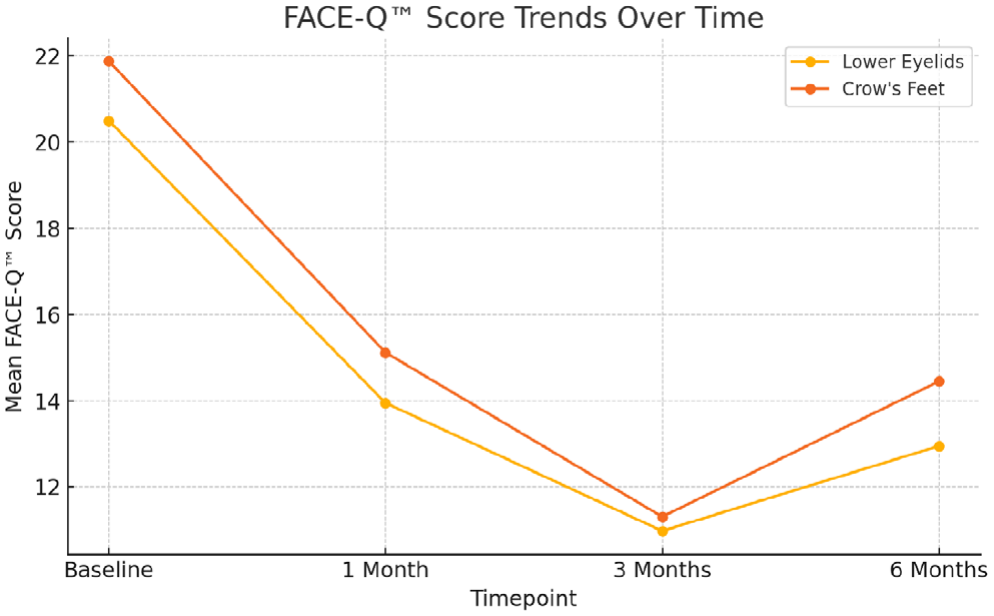
FACE‐Q satisfaction score trends over 6 months.

The greatest improvement occurred at 3 months, following two consecutive treatment sessions, where the mean increase from 1 to 3 months was 4.12 points for Satisfaction with Outcome (95% CI: 3.51–4.73) and 3.83 points for Satisfaction with Decision (95% CI: 3.07–4.60), both *p* < 0.001. Large effect sizes were calculated for appraisal outcomes at 3 months compared with baseline (Cohen's *d* = 6.33 for lower eyelids and 3.51 for crow's feet), indicating substantial within‐subject changes. At 6 months, scores remained significantly improved compared with baseline (*p* < 0.001 for both scales), although a slight decline was noted relative to the 3‐month peak (Figure 1). For appraisal outcomes, mean improvement (baseline minus follow‐up) was 9.52 points for lower eyelids (95% CI: 8.78–10.27) and 10.57 points for crow's feet (95% CI: 9.24–11.90) at 3 months, with sustained improvement at 6 months (lower eyelids: 7.55, 95% CI: 7.11–7.99; crow's feet: 7.43, 95% CI: 6.17–8.69).

**FIGURE 2:**
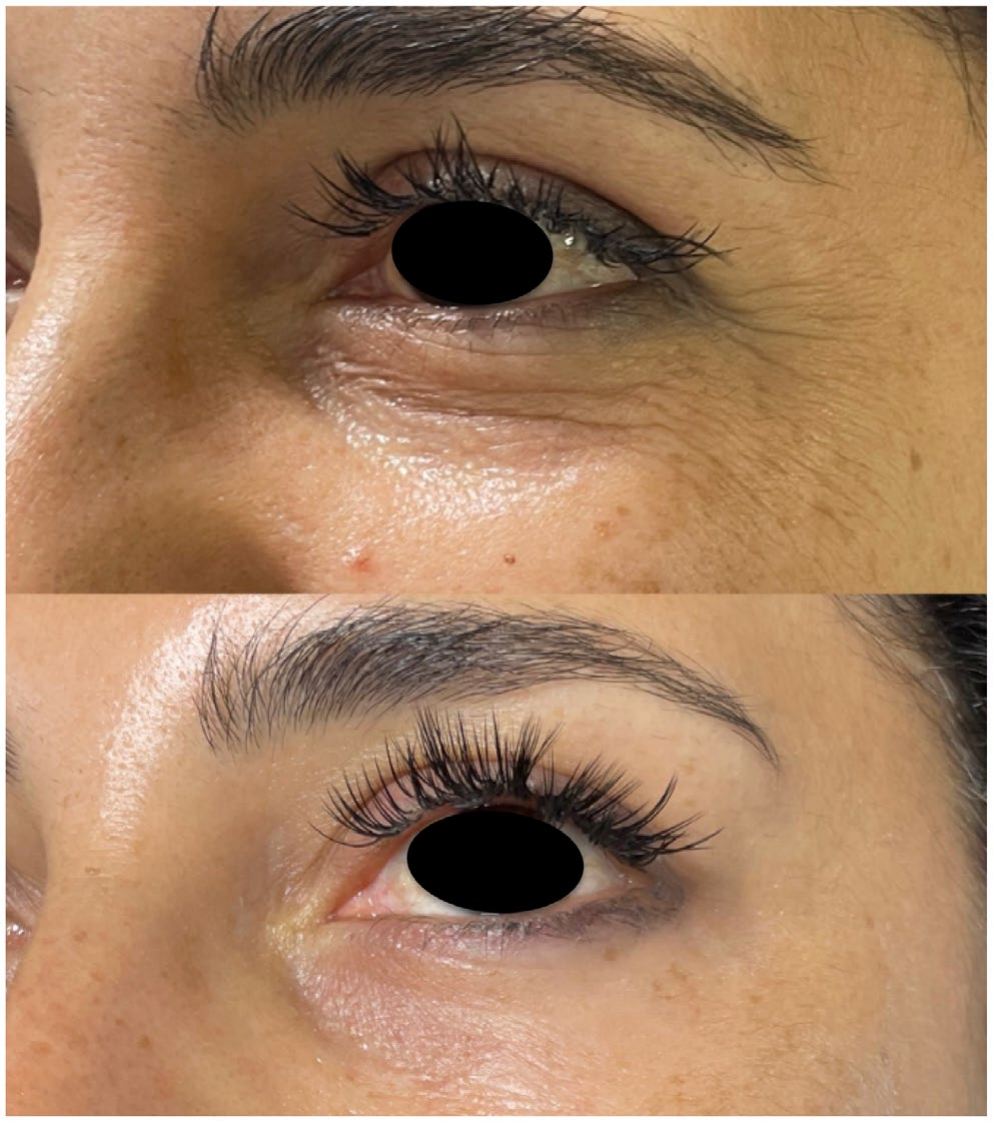
Patient 1. Baseline (top) and 3 months after two PN injection sessions (bottom), showing reduction in infraorbital rhytids and improved skin texture.

**FIGURE 3:**
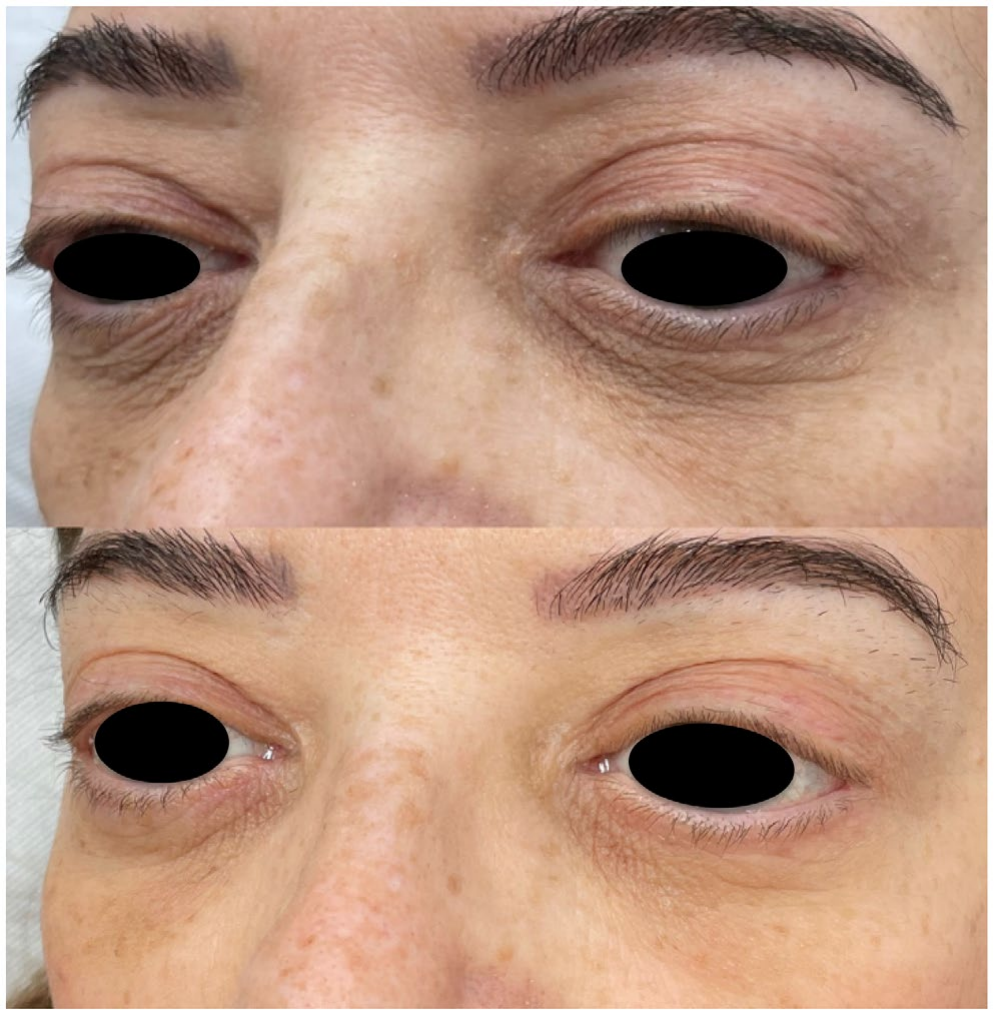
Patient 2. Baseline (top) and 3 months after two PN injection sessions (bottom), demonstrating improved pigmentation, smoother skin surface, and reduction in fine wrinkles.

Across outcomes, the largest subjective improvements were observed at the 3‐month time point following two PN injection sessions. A modest reduction in effect was observed by 6 months, suggesting gradual attenuation of the treatment response and supporting the rationale for maintenance therapy at or before this time point (Figure 1). Representative clinical examples further illustrate these findings, demonstrating improvements in infraorbital skin quality, fine lines, and pigmentation at 3 months post‐treatment (Figures 2 and 3).

## 4 Discussion

Evaluation of lower eyelid and crow's feet appraisal demonstrated improvement beginning after the first treatment session, with effects maintained through 6 months. Across outcomes, changes followed a consistent temporal pattern, characterized by progressive improvement after the first session, maximal response at 3 months following two sessions, and modest attenuation by 6 months. This response trajectory supports further investigation into stratifying patients by baseline severity, with more advanced presentations potentially benefiting from extended induction protocols.

Patient satisfaction with both treatment outcome and decision mirrored this temporal pattern, with highest scores observed at 3 months and sustained improvement at 6 months compared with earlier follow‐up. The parallel changes observed in appraisal and satisfaction measures highlight the clinical relevance of maintenance timing in periorbital rejuvenation.

The findings of this study demonstrate an association between intradermal polynucleotide (PN) injections and improvement in the appearance of undereye wrinkles and crow's feet. Improvement was evident after the first session, which may support patient engagement and adherence, while the most pronounced changes were observed after the second session. This pattern aligns with PN's established role as a biostimulatory agent that induces gradual tissue remodeling rather than immediate volumization.

Our findings are broadly consistent with existing PN literature. Studies by Araco et al. and Abuyousif et al. reported improvements in wrinkle appearance, dermal quality, and periorbital skin hydration following PN‐based treatments [2, 3]. Additional investigations have demonstrated improvements in skin thickness, elasticity, tone, and pigmentation with PN across facial applications [7, 8], while real‐world practice patterns indicate frequent use of PN in the infraorbital region using multi‐session protocols [9]. Expert consensus further supports PN's role in fibroblast stimulation, extracellular matrix remodeling, and anti‐inflammatory activity, recommending induction regimens followed by maintenance treatments [10]. Mechanistic data from prior PDRN/PN research—including adenosine A2A receptor activation, enhanced wound healing, hydrogel‐based hydration, and anti‐melanogenesis effects reported in experimental and clinical studies—provide biological plausibility for these observations [11]. Cavallini et al. likewise demonstrated improvements in elasticity, firmness, smoothness, and brightness following PN‐HPT regimens [12]. Collectively, these studies support PN as a regenerative modality capable of producing sustained improvements in skin quality when delivered intradermally. Importantly, these mechanistic findings derive from studies conducted in various anatomical regions and were not directly assessed in the present periorbital‐focused investigation.

While several PN studies have incorporated objective outcome measures such as imaging, elasticity testing, ultrasound‐based dermal thickness, hydration indices, or melanin quantification, the present study relied primarily on validated patient‐reported outcomes and clinical assessment. Although FACE‐Q instruments are sensitive and widely accepted in aesthetic research, the absence of adjunctive objective endpoints limits direct comparability with recent PN studies and constrains objective characterization of structural tissue changes. This underscores the need for future investigations integrating quantitative assessment modalities alongside patient‐reported outcomes.

Although molecular and histologic mechanisms were not directly evaluated, existing literature provides a biologically plausible framework for interpreting the observed clinical changes. Compared with traditional injectables, PN offers a regenerative rather than paralytic or volumizing approach. Whereas botulinum toxin primarily targets dynamic rhytids and fillers provide volumetric correction with potential risks in the periorbital region, PN is thought to promote fibroblast activity, collagen synthesis, and extracellular matrix remodeling, resulting in gradual, natural‐appearing improvement without added volume [5, 6].

A notable strength of this study is the integration of patient‐reported outcomes with physician assessments, which demonstrated concordant improvement patterns over time. Physician evaluations supported the patient‐reported trajectory, reinforcing the clinical relevance of the findings.

The results informed refinements to the clinical protocol. While two initial sessions were sufficient for most patients, those with more advanced rhytides may benefit from a three‐session induction series followed by maintenance every 4–6 months. The chosen injection technique—multiple intradermal microinjections with a tenting effect—allowed precise dermal delivery and may have enhanced both mechanical and biological stimulation. Prior experience with cannula‐based delivery yielded less favorable outcomes, supporting needle‐based intradermal placement.

The optional third session was offered to a limited subset of patients with persistent moderate‐to‐severe periorbital rhytides following the second session, defined clinically by visible static wrinkles at rest and minimal improvement on appraisal scales. The study was not designed or powered to compare two‐session versus three‐session regimens, and the number of patients receiving a third session was insufficient for meaningful subgroup analysis. Accordingly, no comparative efficacy conclusions are drawn, and recommendations regarding 2–3 initial sessions should be interpreted as experience‐based and hypothesis‐generating.

Overall, highly purified PN was associated with consistent subjective improvement in periorbital rejuvenation and demonstrated a favorable safety profile. No delayed adverse events were identified during longitudinal follow‐up, supporting the tolerability of intradermal PN injections in the periorbital region.

This study was designed to evaluate longitudinal changes within a treated cohort rather than to establish causal efficacy. As an uncontrolled prospective case series relying primarily on patient‐reported outcomes and non‐blinded physician assessments, the findings are susceptible to regression to the mean, placebo effects, and expectation bias. Within the hierarchy of evidence, these results should be interpreted as hypothesis‐generating signals warranting confirmation in controlled studies.

Several limitations must be acknowledged. The exploratory design precluded formal sample‐size calculation, objective imaging modalities were not incorporated, and multiple comparisons were performed without correction, increasing the potential for type I error. The absence of a control group and evaluator blinding further limits causal inference. In addition, all procedures were performed at a single private clinic by one injector, which may limit reproducibility and generalizability and could introduce center‐ or operator‐specific influences on patient‐reported outcomes. Future multicenter randomized studies incorporating objective assessment tools, blinded evaluation, and prespecified statistical plans are needed to confirm these findings and refine treatment protocols.

## 5 Conclusion

In this prospective observational case series, intradermal polynucleotide injections were associated with improvements in patient‐reported appraisal and satisfaction related to undereye wrinkles and crow's feet. The treatment was generally well tolerated within this cohort, with minimal adverse events observed during follow‐up. Improvements followed a consistent temporal pattern, with maximal response after two treatment sessions and gradual attenuation over time. A treatment approach involving two to three initial sessions with subsequent maintenance based on wrinkle severity reflects clinical experience but should be interpreted as hypothesis‐generating. Future controlled studies incorporating comparator groups and objective outcome measures are needed to confirm efficacy and further refine treatment protocols.

## Author Contributions

G.Z. conceived and designed the study. G.Z. and E.K. contributed to manuscript writing and study coordination. E.K., J.E.M., and D.S. contributed to data analysis and interpretation. D.D. performed the statistical analysis and provided methodological support. G.F. provided senior dermatologic oversight and critically reviewed the manuscript. All authors contributed to manuscript revision, read, and approved the final manuscript.

## Funding

The authors have nothing to report.

## Conflicts of Interest

The authors declare no conflicts of interest.

## Data Availability

The data that support the findings of this study are available from the corresponding author upon reasonable request.

## Appendix A Polynucleotides Questionnaire Before and After Undereye Treatment

Patient number:

Initials:

Age:

Diagnosis/Grade:

### Before Treatment

- FACE‐QTM—SATISFACTION WITH SKIN
- 1 Very dissatisfied 4 Very satisfied
- How your facial skin looks at the end of your day?
- How healthy does your skin looks?
- How attractive does your facial skin make you look?
- How smooth your skin looks?
- How clear your skin looks>
- How refreshed your skin makes you look?
- How hydrated?
- How it looks when you wake up?
- How radiant it looks?
- How the color looks?
- How your pores look?
- How even colored it looks?

### One Month After Treatment

- FACE‐QTM—SATISFACTION WITH SKIN
- 1 Very dissatisfied 4 Very satisfied
- How your facial skin looks at the end of your day?
- How healthy does your skin looks?
- How attractive does your facial skin make you look?
- How smooth your skin looks?
- How clear your skin looks>
- How refreshed your skin makes you look?
- How hydrated?
- How it looks when you wake up?
- How radiant it looks?
- How the color looks?
- How your pores look?
- How even colored does it looks?

### Five Months After Treatment

- FACE‐QTM—SATISFACTION WITH SKIN
- 1 Very dissatisfied 4 Very satisfied
- How your facial skin looks at the end of your day?
- How healthy does your skin looks?
- How attractive does your facial skin make you look?
- How smooth your skin looks?
- How clear your skin looks>
- How refreshed does your skin make you look?
- How hydrated?
- How it looks when you wake up?
- How radiant it looks?
- How the color looks?
- How your pores look?
- How even colored it looks?

### Physician 1 at 1 Month

- 7‐Point Global Assessment Scale
- Significant deterioration −3
- Moderate deterioration −2
- Slight deterioration −1
- No change 0
- Slight improvement +1
- Moderate improvement +2
- Significant improvement +3
- Score:

### Physician 2 at 5 Months

- 7‐Point Global Assessment Scale
- Significant deterioration −3
- Moderate deterioration −2
- Slight deterioration −1
- No change 0
- Slight improvement +1
- Moderate improvement +2
- Significant improvement +3
- Score:

## References

[1] A. Samadi , A. Ayatollahi , M. Nassiri‐Kashani , et al., “Efficacy and Tolerability of a Polynucleotide‐Based Gel for the Improvement of Pattern Hair Loss,” Archives of Dermatological Research 316 (2024): 331. 38842633 10.1007/s00403-024-03088-9

[2] H. S. Abuyousif , A. Porcello , M. Cerrano , et al., “In Vitro Evaluation and Clinical Effects of a Regenerative Complex With Non–Cross‐Linked Hyaluronic Acid and High‐Molecular‐Weight Polynucleotide for Periorbital Treatment,” Polymers 17, no. 5 (2025): 638, 10.3390/polym17050638. 40076130 PMC11902836

[3] A. Araco , F. Araco , and M. Raichi , “Clinical Efficacy and Safety of Polynucleotides Highly Purified Technology (PN‐HPT) Combined With Cross‐Linked Hyaluronic Acid for Moderate to Severe Nasolabial Folds: A Prospective Randomized Exploratory Study,” Journal of Cosmetic Dermatology 22, no. 1 (2023): 146–155, 10.1111/jocd.15064. 35531796 PMC10084116

[4] A. Araco and F. Araco , “A Prospective Randomized Study of Highly Purified Polynucleotides Versus Placebo in the Treatment of Moderate to Severe Acne Scars,” Aesthetic Surgery Journal 41 (2021): NP866–NP874. 33755110 10.1093/asj/sjab125

[5] M. Cavallini , E. Bartoletti , L. Maioli , et al., “Consensus Report on the Use of Polynucleotides Highly Purified Technology (PN‐HPT) in Aesthetic Medicine,” Journal of Cosmetic Dermatology 20 (2021): 922–928. 32799391 10.1111/jocd.13679 PMC7984045

[6] K. W. A. Lee , K. W. L. Chan , A. Lee , et al., “Polynucleotides in Aesthetic Medicine: Current Practices and Perceived Effectiveness,” International Journal of Molecular Sciences 25, no. 15 (2024): 8224. 39125793 10.3390/ijms25158224 PMC11311621

[7] K. Y. Park , J. Y. Lee , J. Y. Kim , et al., “Long‐Chain Polynucleotide Filler for Skin Rejuvenation: Efficacy and Complications in Five Patients,” Dermatologic Therapy 29, no. 1 (2016): 37–40, 10.1111/dth.12299. 26814448

[8] Y. J. Lee , W. S. Kim , J. H. Lee , et al., “Comparison of Polynucleotide and Hyaluronic Acid Fillers for Periocular Rejuvenation: A Randomized Double‐Blind Split‐Face Trial,” Journal of Dermatological Treatment 33, no. 1 (2022): 254–260, 10.1080/09546634.2020.1748857. 32248707

[9] N. K. Rho , J. H. Kim , S. Y. Park , et al., “Survey on Cosmetic Use of Injectable Polynucleotides: Patterns of Practice Among Korean Dermatologists,” Journal of Cosmetic Dermatology 23, no. 4 (2024): 1243–1252, 10.1111/jocd.16125. 38093498

[10] K. Aawrish , W. Guobao , Z. Feng , et al., “Polydeoxyribonucleotide as a Promising Skin Anti‐Aging Agent,” Chinese Journal of Plastic and Reconstructive Surgery 4, no. 4 (2022): 187–193.

[11] T. K. Noh , S. Y. Lee , J. H. Kim , et al., “Anti‐Melanogenesis Properties of Polydeoxyribonucleotide in Experimental Models,” International Journal of Molecular Sciences 17, no. 9 (2016): 1448, 10.3390/ijms17091448. 27598132 PMC5037727

[12] M. Cavallini , C. De Luca , G. Prussia , and M. Raichi , “PN‐HPT in Rejuvenation of the Facial Middle Third: Clinical Outcomes and Patient Satisfaction,” Journal of Cosmetic Dermatology 21, no. 2 (2022): 615–624, 10.1111/jocd.14578. 34791770 PMC9299481

